# *Dermanyssus gallinae*: the long journey of the poultry red mite to become a vector

**DOI:** 10.1186/s13071-021-05142-1

**Published:** 2022-01-20

**Authors:** Antonella Schiavone, Nicola Pugliese, Domenico Otranto, Rossella Samarelli, Elena Circella, Caterina De Virgilio, Antonio Camarda

**Affiliations:** 1 Department of Veterinary Medicine, University of Bari “Aldo Moro”, Valenzano, Italy; 2 Department of Biosciences, Biotechnologies and Biopharmaceutics, University of Bari “Aldo Moro”, Bari, Italy

**Keywords:** *Dermanyssus gallinae*, Vectorial role, Pathogens, Poultry red mite, Bacteria, Viruses

## Abstract

The possibility that *Dermanyssus gallinae*, the poultry red mite, could act as a vector of infectious disease-causing pathogens has always intrigued researchers and worried commercial chicken farmers, as has its ubiquitous distribution. For decades, studies have been carried out which suggest that there is an association between a wide range of pathogens and *D. gallinae*, with the transmission of some of these pathogens mediated by *D. gallinae* as vector. The latter include the avian pathogenic *Escherichia coli* (APEC), *Salmonella enterica* serovars Enteritidis and Gallinarum and influenza virus. Several approaches have been adopted to investigate the relationship between *D. gallinae* and pathogens. In this comprehensive review, we critically describe available strategies and methods currently available for conducting trials, as well as outcomes, analyzing their possible strengths and weaknesses, with the aim to provide researchers with useful tools for correctly approach the study of the vectorial role of *D. gallinae*.

**Graphical Abstract:**

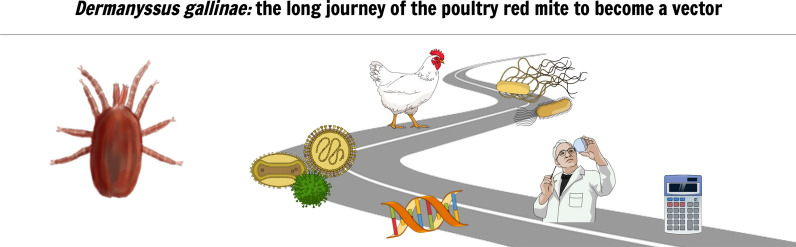

## Background

The poultry production system considers infestation by *Dermanyssus gallinae*, commonly referred to as the poultry red mite (PRM), to be a matter of concern because of its deleterious impact on both productivity and welfare of the animals [1, 2]. In addition, in highly infested environments, poultry workers may also be at risk of infestation by PRMs, with clinical conditions varying from itching, dermatitis and erythematous rashes to papules, urticarial plaques and erythema [3–7]. The control strategies mostly rely on the application of synthetic or semisynthetic acaricides [8], with phoxim, fluralaner and spinosad being among the few acaricides authorized worldwide [9]. However, the development of mite populations resistant to these chemical compounds has often led farmers to use unauthorized drugs, such as amitraz or fipronil, which could be harmful to human health [10].

Due to its specific biology and blood-feeding habits on birds, the pathogenic effect exerted by PRMs has major consequences for the poultry industry, potentially playing a role in spreading infectious disease-causing pathogens [11]. The role of the PRM as a vector of pathogens has been investigated since the mid-1940s, beginning in a series of experimental infections with pathogens, such as the St. Louis encephalitis virus and the equine encephalomyelitis virus [12, 13]. Subsequent studies focused specifically on poultry-specific diseases, such as fowl spirochetosis [14] or fowl cholera [15]. Ultimately, a wide range of pathogens has been associated with *D. gallinae* infestation and, in some cases, PRM-mediated transmission has also been demonstrated (Table 1). However, current knowledge on the role of the PRM as a vector of pathogens is far from complete, also because of the lack of harmonized procedures for conducting these kinds of studies. Recently, that objective has been addressed by the EU Cooperation in Science and Technology (COST) Action FA1404 “Improving current understanding and research for sustainable control of the poultry red mite *Dermanyssus gallinae*” [16]. Therefore, the aim of this review is to critically analyze the current state of knowledge on the interactions between *D. gallinae* and potential pathogens, with attention also paid to the new strategies and tools available for conducting robust trials on this important subject.

**Table 1:** Pathogens detected in *Dermanyssus gallinae* mites or for which the mite-mediated transmission has been demonstrated

Kingdom	Pathogen	Described relation with *D. gallinae*	Reference
Bacteria	*Salmonella* Gallinarum	Transmission demonstrated	[18]
	*Escherichia coli*	Detected in *D. gallinae*	[36]
	*Tsukamurella*	Detected in *D. gallinae*	[42]
	*Mycoplasma synoviae*	Detected in *D. gallinae*	[40]
	*Mycoplasma gallisepticum*	Detected in *D. gallinae*	[40]
	*Chlamydia psittaci*	Detected in *D. gallinae*	[38]
	*Salmonella* Enteritidis	Transmission demonstrated	[77]
	*Erysipelothrix rhusiopathiae*	Detected in *D. gallinae*	[73]
	*Pasteurella multocida*	Transmission demonstrated	[15]
	*Borrelia burgdorferi*	Detected in *D. gallinae*	[39]
	*Coxiella burnetii*	Transmission demonstrated	[39]
	Spirochetes	Transmission demonstrated	[97]
Viruses	Newcastle disease virus	Detected in *D. gallinae*	[19]
	Avian influenza virus	Transmission demonstrated	[17]
	Fowl poxvirus	Transmission demonstrated	[47]
	Saint-Louis encephalitis virus	Detected in *D. gallinae*	[12]
	Tick-borne encephalitis virus	Detected in *D. gallinae*	[98]
	Western equine encephalitis virus	Transmission demonstrated	[31]
	Venezuelan equine encephalitis virus	Transmission demonstrated	[32]
Protozoa	*Plasmodium* sp.	Detected in *D. gallinae*	[41]

## Inaccessibility and misinterpretation of some experimental studies: literature or legend?

The scientific literature on the relationship between *D. gallinae* and pathogens they may carry is quite variable. Some papers describe rigorous experimental studies that are discussed with clarity and objectiveness [17, 18]. Others are quite controversial in their interpretation or simply inaccessible to many scientists because they are written in the local language. The latter is the case of papers describing the association of PRMs and *Borrelia anserina* [14], and of PRMs and *Pasteurella multocida* [15], written in Russian and Bulgarian, respectively. Furthermore, many studies are not available online, resulting in them being unaccessible to most researchers interested in the subject. For example, the association between the PRM and Newcastle disease virus (NDV) is based on a paper by Arzey [19] that was published in 1990 as a bulletin in a government series of the Department of Agriculture of South Wales (Australia), in which the author refers to a 1964 report [20] published as an abstract. Other authors have referred to the association between *D. gallinae* and mycobacteria [21] based on a 2007 PhD thesis [22] that reports positive PCR and RT-PCR tests only from mite samples when using *Mycobacterium*-specific* 16S* primers but negative results with 13.8-kDa bacteriolytic enzyme amplification. The results described here are clearly controversial but, over time, have been used to confirm the vectorial role of PRMs for mycobacteria [21], a conclusion disputed even by the same authors in the Discussion section.

Other cases of deceiving citations are due to a kind of “Chinese whispers” game from author to author. A putative association between *D. gallinae* and some protozoa and filariae has been reported in several papers [4, 23]. Nonetheless, going back to the origin of this information, Valiente Moro et al. [24] claimed in their review that the potential relationship of those pathogens was with *Ornithonyssus bacoti* [25, 26] and not *D. gallinae*. The latter was incorrectly considered for long time as being based on scientific evidence.

## The PRM as a vector: To be or not to be?

For *D. gallinae* to be considered a competent vector of pathogen(s), it needs to fulfill the requirements of the definition of a vector (i.e. an arthropod capable of transmitting a pathogen to vertebrate hosts [27]) that will eventually disseminate a pathogen-specific infection [28]. The concepts described in the preceding text are based on the ability of *D. gallinae* to become infected and transmit a microorganism (i.e. vector competence), a feature that, in turn, is based on the biological traits of both pathogens and vectors [29]. Following the assessment of an arthropod as a vector of a given pathogen, the modalities of its transmission (i.e. mechanical or biological) should be demonstrated. Only a few studies have described the pathogen transmission modalities for *D. gallinae*. In early studies, chickens infected with the Saint Louis encephalitis virus were exposed to PRMs and then subsequently put in contact with a group of uninfected chickens; viremia in the latter group was then assessed by inoculating mice with their blood [30]. A similar approach was used to study the PRM-mediated transmission of Western equine encephalitis virus [31]. The involvement of PRMs in the transmission of Eastern and Venezuelan equine encephalitis viruses was studied by plaque assay and plaque-reduction neutralization tests [32, 33]. Although the mite-mediated transmission of the Venezuelan equine encephalitis virus has been demonstrated among mice and not birds [32], the vectorial role of PRMs for that virus is inappropriately referred to in several papers [21, 24, 34, 35].

Recently, PRM-mediated transmission was proven for the avian influenza A virus in specific-pathogen-free (SPF) chickens following contact of the latter with chickens infected with *D. gallinae* [17]. This study also demonstrated that mites act as mechanical vectors for the infection through their ingestion. The competence of *D. gallinae* as a vector of *Salmonella enterica* subsp. *enterica* serovar Gallinarum (*S*. Gallinarum; i.e. the causative agent of fowl typhoid) has been assessed in an elegant trial conducted using isolators [18], in which entomological parameters (e.g. vector capacity) were established to be even higher than those of mosquitoes. Nonetheless, more specific algorithms should be set up for assessing the vectorial role of mites, given their peculiarities in terms of biology and population dynamics. One study applied a strategy based on both molecular and cultural techniques to assess the association between *D. gallinae* and the avian pathogenic *Escherichia coli* (APEC) [36]. A series of specific PCR and quantitative (q)PCR analyses successfully detected and quantified *E. coli* in mites, also discriminating among commensal and potential pathogenic serogroups. Most studies have reported similar associations between *D. gallinae* and specific pathogens. In some cases, the same pathogen detected in PRMs was also found to infect parasitized animals, such as the cases of *Erysipelothrix rhusiopathiae* [37], *Chlamydia psittaci* [38] or *E. coli* [36] while, in contrast, *Borrelia burgdorferi* sensu lato, *Coxiella burnetii* [39], *Mycoplasma gallisepticum*, *Mycoplasma sinoviae* [40], *Plasmodium* sp. [41] and *Tsukamurella* sp. [42] were only found in the mites but not in the hosts.

The presence of some of the above microorganisms exclusively in *D. gallinae* could imply its role as a reservoir (i.e. a host that can maintain the pathogen for a prolonged time, ref. [43]), although little is known about the persistence of these microorganisms in mites**.** For example, it was estimated that *D. gallinae* may host *S.* Gallinarum for up to 4 months [44]. Anecdotical information [34, 45, 46] suggests that *P. multocida* [15] and avian poxvirus [47] may persist in PRMs up to 64 and 300 days, respectively, although the original papers are among those not available in the international literature. More recently, Pugliese et al. studied a fowl typhoid outbreak and found *S.* Gallinarum in fowl tested during a sanitary break, which had been absent in the animals for 2 months, in association with *D. gallinae* [48]. Considering that *S*. Gallinarum was circulating once again in the new flock (i.e. 5 months apart), the authors suggested that *D. gallinae* might have acted as a reservoir, reintroducing the pathogen into the newly housed animals.

## Microbiome of the PRM

The pathogenic nature of *S.* Gallinarum has greatly facilitated the establishment of a relationship between *D. gallinae* and fowl typhoid. However, *D. gallinae* can habor many other microorganisms, and refined studies are needed to demonstrate their role as pathogens. Among these, *E. coli* species includes both commensal and pathogenic strains of *D. gallinae* [49], which may be carried simultaneously by mites [36], making their identification essential. Again, considering the complex genetic profiles conferring pathogenicity to the *E. coli* strains [50], the isolation of the pathogen could be critical to selecting and delineating the potentially pathogenic strains within the heterogeneous population of *E. coli* hosted by *D. gallinae*. Therefore, once isolated, determination of the *E. coli* virulence profile is imperative to identify them as the same strains isolated from animals. Determination of the virulence profile has been achieved using different approaches. The first approach consisted of separate PCRs targeting the shared *uidA* gene (encoding β-glucuronidase) and the genetic determinants of serogroups to reveal the presence of *E. coli* and, more specifically, of APEC [36]. A second approach focused on the characterization of three different pathogenicity genes of *E. coli* (i.e. *neuS*, *tsh*, *iss*) and the housekeeping gene *phoA* as a species marker [46].

Conceivably, the metagenomics approaches have limitations due to the opportunistic or pathogenic nature of bacterial agents. An investigation based on* 16S* RNA PCR amplification revealed the presence of operational taxonomic units that may include potentially pathogenic bacteria, such as *Bartonella* sp., *Staphylococcus* sp. or *Tsukamurella* sp., but no data on virulence was retrieved by characterizing rRNA genes [42, 51, 52]. However, the –omic surveys disclosed a wide number of environmental, commensal and symbiotic bacteria, such as *Bartonella*-like symbionts, associated with *D. gallinae* [42, 53, 54], highlighting a degree of variation in the microbiome composition according to the life-cycle stages and location of mites; these may represent important factors to consider in planning investigations about PRMs and microorganisms. Analogously, it cannot be ruled out that the mite microbiome may be affected in some degrees by the farm microenvironment, which could create the conditions for the colonization and persistence of pathogens. Thus, it should also be considered that the PRM may carry pathogen-vaccine strains. Indeed, while live attenuated vaccines against viral and bacterial pathogens are commonly used in poultry farming [55, 56], they might be present in the environment and, therefore, in mites. Vaccine strains of the avipox virus, *Mycoplasma synoviae* and *M. gallisepticum* have been detected in *D. gallinae* [40], potentially competing with the pathogenic ones and contributing to a reduction in the circulation of the wild-type strains [57]. Nevertheless, the persistence of vaccine strains could lead to some drawbacks to the flock, such as the spread to unvaccinated groups or excessive immunologic pressure [57]. Furthermore, this could cast doubts on the nature of the pathogen (i.e. vaccine or wild type), further complicating the diagnosis for some diseases [58]. Finally, the coexistence of both vaccine and wild-type strains might promote the upsurge of new strains of pathogens through recombination [57]. From this perspective, on a merely hypothetical basis, *D. gallinae* might act as a melting pot where different strains come in close contact, fostering recombination events.

## PRM blood-feeding: the first step toward pathogen acquisition

Demonstration of the vectorial role of PRMs must be corroborated by specific data and information. As a first step, mites must come in contact with the host and, more specifically, with the biological parts infected by the pathogen. The most effective strategy to demonstrate the vectorial role of PRMs is to experimentally infest animals; however, this approach is limited due to ethical, economic and bureaucratic concerns, which in turn are enhanced by the need for isolators and SPF animals, and by the necessity to infest the animal with thousands of mites. The latter condition makes population monitoring more difficult, especially in terms of life-cycle stage and reproductive fitness [59]. To overcome some of these issues, mites have been put in contact with chickens by including them in a small pouch made of nylon phytoplankton mesh attached to the bird skin [60]. In addition to the evident advantages from a welfare perspective, this method may be considered an artificial in vivo device that allows the harvesting of mites after the blood meal. Additionally, the method limits the dispersal of mites, which can all be collected after the feeding experiment. However, despite the effectiveness of this method, researchers still need to deal with live animals. Therefore, several in vitro feeding devices have been developed and used in various studies, based on both biological and artificial membranes, essentially replicating protocols already in use for ticks (which mainly employ synthetic membranes [61]). However, as yet optimal devices for studies with *D. gallinae* are not yet available because of the significant differences in the size and anatomy of mouthparts [62]. The tick hypostome bears denticles for anchoring in the host’s skin, which are absent in mites. In addition, ticks are slow feeders and may take several days to engage and, therefore, need an anchoring system [63], which is lacking in mites that carry a claw-like structure, also referred to as apotele or palpal claw [64].

The first attempts to feed PRMs with an artificial system were carried out using 1-day-old chick skins [59, 65], which were more effective than synthetic membranes: a comparative study demonstrated a feeding rate of about 39% of mites from devices with chick skin, and between 5 and 32% from the same devices with Nescofilm or rayon membranes [66]. Therefore, by using a chick skin system, it was possible to demonstrate effective infection of mites with *S.* Enteritidis through contaminated blood meals as well as the vertical transmission of the pathogen to the next generation [65]. More recently, an effective alternative to the 1-day-old chick skin was described by Nunn et al. [67], who found that about 50% of mites fed from a device consisting of goose blood as a food source and goldbeater’s skin membrane derived from bovine intestine (known as Baudruche), commonly used for repairing ancient manuscript due to its thickness and resistance. Such material is commercially available and is inexpensive, and therefore removes the need to sacrifice 1-day-old chicks for specific research purposes.

In addition to infecting animals with microorganisms through blood-feeding, another modality could be mechanical contamination through adherence to the mites (e.g. dorsal and ventral shields, the legs, mouthparts). Many pathogens are excreted by birds in their feces [68] or shed in respiratory secretions [69, 70] or skin exfoliation [71]; thus, mites may be exposed to these pathogens by their movements through contaminated animal shelters [72]. The invasion of mites by microorganisms during their off-host life-cycle stages has already been suggested, hypothesizing the introduction of pathogens through transcuticular absorption or the respiratory system [65]. For these reasons, most researchers have focused their efforts on assessing the internal presence of the pathogens by washing mites with 4% paraformaldehyde in order to remove the surface-contaminating microorganisms [18, 36, 44, 65].

## A matter of time: pathogen incubation periods in the PRM

Knowledge of pathogen incubation time is crucial for choosing the most suitable time for putting mites on the hosts in order to enhance the chances for them to acquire infections. Ideally, this should occur when the pathogen is spreading through the host’s bloodstream. An explicative example is represented by experimental infection with *Eysipelothrix rhusiopathiae* [73]. When mites were introduced into the isolators the day after experimental infection of the host with the bacteria, all of them tested negative after 6 days, even though birds developed clinical signs 3 days post-infection. These data suggest that the pathogen could not have yet invaded the bloodstream of the hosts when mites had their blood meal. Accordingly, when hens have been infested with *D. gallinae* 7 days after being experimentally infected by *S.* Gallinarum, a high percentage of positive mites (87.5%) was recorded [18], in accordance with the peak of bacteriemia, which occurs after 7 days [74].

Additionally, the starvation of mites may be pivotal for the acquisition of the infection, in that the complete digestion of a previous blood meal enhances their aggressiveness and host-seeking behavior [75]. Hence, the feeding rate is higher after 2–3 days (60%) and 8–10 days (75%) from the last blood meal [72], while it drops after 14–16 days of starvation, this period being too long for maintaining mite vital functions [75]. Temperature has also been found to be a conditioning factor as well; feeding rate was found to be high for mites kept at room temperature for 7 days, and even higher for those starved and cooled at 5 ± 1 °C for 30 days [76]. However, a significant reduction in feeding rate was observed in a separate experiment when adults were starved for 2 weeks at 4 °C [60].

## Possible pathways of pathogen transmission by the PRM

Few studies have established the route of the mite-mediated infection of the host, despite this being a relevant issue [77]. The majority of hematophagous vectors, like ticks and mosquitos, usually transmit pathogens when biting hosts [63, 78, 79]. Tick-transmitted pathogens represent an example, since, following their consumption with blood, they migrate from the gut to the hemocoel, reaching the salivary glands and eventually being transmitted to another host with the next blood meal [80]. Thus, the colonization of salivary glands is a necessary step for these arthropods being competent vectors [81].

The oral entry route of pathogens in the host through the ingestion of infected mites has been described only sporadically [17, 77]. The in vivo artificial feeding devices [60, 82] allow infected mites to infest the host without being pecked and ingested by birds. Finally, invasion of the ovaries and ovarian germinal tissue of mites should not be neglected, as it might represent another way for pathogens to spread to the mite progeny (i.e. vertical transmission). To date, transovarial and transstadial pathways have been proven for *S.* Enteritidis in *D. gallinae* [65], although the modalities of bacterial colonization of mites is still unknown. However, although knowledge of the anatomy and biology of *D. gallinae* has improved in recent years [83–85], the localization of pathogens is still unkown due to difficulties in dissecting mites [86, 87]. Conversely, data are available on pathogen localization in ticks through fluorescent in situ hybridization [88], direct immunofluorescence and/or immunohistochemistry [89]. Therefore the scientific know-how on ticks could be a starting point for further study on the occurrence and the biology of pathogens in PRMs.

## Pathogen infection in the PRM: quantitative data analysis

To our knowledge, very little quantitative data have been produced to date on the vector potential of *D. gallinae*. Most of the earlier studies were based on qualitative data derived from the detection of pathogens from mites by in vitro culture methods. The introduction of molecular tools (e.g. PCR, qPCR) has provided refined opportunities for yielding quantitative data. Nonetheless one of the limitations to these latter approaches is the processing of mites singularly, so that detection of pathogens is usually performed from pools, which makes the assessment of mite infection rate more difficult. Recently, a PooledInfRate tool [90], originally used to estimate the minimum infection rate in ticks and mosquitoes [91–93], was used for PRMs [18, 36, 48]. In addition, while ticks pools are usually composed of about five to six specimens [88, 91, 94], a larger number of individuals is required for studies on *D. gallinae* (50–100 individuals), implying the adoption of wider confidence intervals with some limitation in the power of the statistical analysis [18]. Notwithstanding, the only available data on mite infection rate for 1000 *D. gallinae* range between 13.72 and 55.21 for *S.* Gallinarum [18] and 24.39 for *E. coli* [36] infected mites, respectively.

Similarly, the application of qPCR strategies has allowed quantitative data to be obtained on the pathogen load of *D. gallinae*, with large variability reported for the load of *S*. Gallinarum per mite (from 5.25 to 629.05 cells/mite [18]) and *E. coli* O2 (from 0.55 to 3789.26 cells/mite [36]**)**. In both cases, the large intervals reflected a stochastic distribution of bacterial cells in mites which limits the use of tools devised to analyze normally distributed data (e.g. mean, standard deviation, Student’s t-test or ANOVA). Therefore, more suitable tools have been used, such as the Hodges-Lehmann location estimator for a more ductile estimation of the central value [95] to be used instead of median, or the Mann–Whitney U-test, a nonparametric test for the comparison of datasets with non-normal distribution [96].

## Conclusions

The pioneering studies on the role of *D. gallinae* as a vector of pathogens were carried out in the 1940s, and in the following decades increasingly more information relevant to this issue has been acquired. Nonetheless, data in the literature are far fom being exhaustive, and there is still large room for improvement through specific investigations aimed at answering crucial questions. Reasoned confirmatory studies are necessary for assessing some controversial associations between the PRM and pathogens, which appear to lack substance in literature, such as in the case of *Mycobacterium* spp., NDV, fowlpox virus or *P. multocida*.

Further developments could be achieved for those well-established associations but for which details are still not ascertained, such as in the case of some aspects of the PRM–pathogen relationship (e.g. survival or replication of microorganisms in mites, the route of the mite-mediated infection of the host, the localization of pathogens within the mites and the pathogen vertical transmission) that still need to be carefully assessed. The introduction of new technologies and, consequently, new approaches may facilitate such studies. New methods have been devised for the in vitro culture of mites and innovative molecular tools have been designed. However, these still need to be tailored for *D. gallinae*, along with new mathematical and statistical models, in order to have more robust treatments of data.

Also, the creation of stable research networks [16] is required for the harmonization of strategies and techniques, which may be based on standard operational procedures that should be fine-tuned to fit the physiology and behavior of *D. gallinae*.

Definitely, a lot still has to be done: the journey of PRM to be considered as a vector is still ongoing.

## Data Availability

Not applicable.

## Abbreviations

APEC: Avian pathogenic *Escherichia coli*
NDV: Newcastle disease virus
PRM: Poultry red mite
*S. *Gallinarum: *Salmonella enterica* Subsp. *enterica* (*S.*) ser. Gallinarum
SPF: Specific-pathogen-free
